# 2-Trifluoro­methyl-1*H*-benzimidazol-3-ium perchlorate

**DOI:** 10.1107/S1600536811039298

**Published:** 2011-10-05

**Authors:** Ming-Liang Liu

**Affiliations:** a College of Chemistry and Chemical Engineering, Southeast University, Nanjing 210096, People’s Republic of China

## Abstract

In the title salt, C_8_H_6_F_3_N_2_^+^·ClO_4_^−^, the atoms of the benzimidazole ring (including H atoms) are nearly coplanar (r.m.s. deviation of the fitted atoms = 0.0122 Å) and the triflouromethyl group lies out of this plane. The perchlorate anion adopts a distorted tetra­hedral conformation with the Cl—O bond distances ranging from 1.412 (3) to 1.439 (2) Å. The benzimidazolium cations are linked to adjacent anions by inter­molecular N—H⋯O hydrogen bonds, forming chains.

## Related literature

For background to mol­ecular–ionic compounds, see: Yu *et al.* (2004 ▶); Chen *et al.* (2009 ▶); Ge *et al.* (2007 ▶).
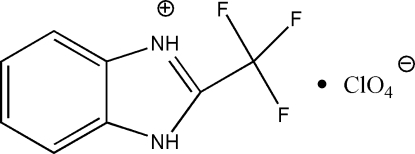


## Experimental

### 

#### Crystal data

C_8_H_6_F_3_N_2_^+^·ClO_4_^−^
*M* *_r_* = 286.60
Triclinic, 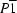

*a* = 7.6274 (15) Å
*b* = 9.0614 (18) Å
*c* = 9.3838 (19) Å
α = 61.80 (3)°
β = 81.98 (3)°
γ = 75.85 (3)°
*V* = 554.0 (3) Å^3^
*Z* = 2
Mo *K*α radiation
μ = 0.40 mm^−1^
*T* = 293 K
0.36 × 0.32 × 0.28 mm

#### Data collection

Rigaku Mercury2 diffractometer
Absorption correction: multi-scan (*CrystalClear*; Rigaku, 2005 ▶) *T*_min_ = 0.963, *T*_max_ = 0.971
5756 measured reflections
2535 independent reflections
1980 reflections with *I* > 2σ(*I*)
*R*_int_ = 0.026

#### Refinement

*R*[*F* ^2^ > 2σ(*F* ^2^)] = 0.051
*wR*(*F* ^2^) = 0.140
*S* = 1.04
2535 reflections
163 parameters
H-atom parameters constrained
Δρ_max_ = 0.49 e Å^−3^
Δρ_min_ = −0.34 e Å^−3^

### 

Data collection: *CrystalClear* (Rigaku, 2005 ▶); cell refinement: *CrystalClear*; data reduction: *CrystalClear*; program(s) used to solve structure: *SHELXS97* (Sheldrick, 2008 ▶); program(s) used to refine structure: *SHELXL97* (Sheldrick, 2008 ▶); molecular graphics: *SHELXTL* (Sheldrick, 2008 ▶) and *PLATON* (Spek, 2009 ▶); software used to prepare material for publication: *SHELXTL*.

## Supplementary Material

Crystal structure: contains datablock(s) I, global. DOI: 10.1107/S1600536811039298/go2027sup1.cif

Structure factors: contains datablock(s) I. DOI: 10.1107/S1600536811039298/go2027Isup2.hkl

Supplementary material file. DOI: 10.1107/S1600536811039298/go2027Isup3.cml

Additional supplementary materials:  crystallographic information; 3D view; checkCIF report

## Figures and Tables

**Table 1:** Hydrogen-bond geometry (Å, °)

*D*—H⋯*A*	*D*—H	H⋯*A*	*D*⋯*A*	*D*—H⋯*A*
N1—H1*A*⋯O2	0.86	2.05	2.891 (3)	164
N1—H1*A*⋯O3	0.86	2.59	3.254 (4)	135
N2—H2*A*⋯O1^i^	0.86	1.98	2.822 (3)	167

Symmetry code: (i) 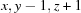.

**Fig. 1.**
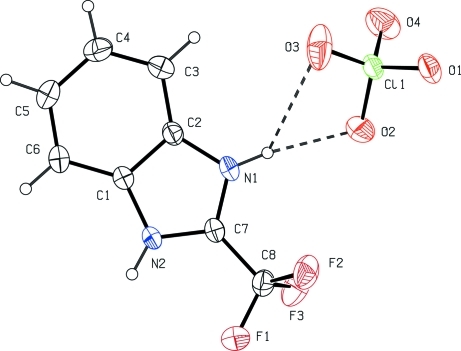
A view of (I) with the numbering scheme. Displacement ellipsoids are drawn at the 30% probability level.

**Fig. 2.**
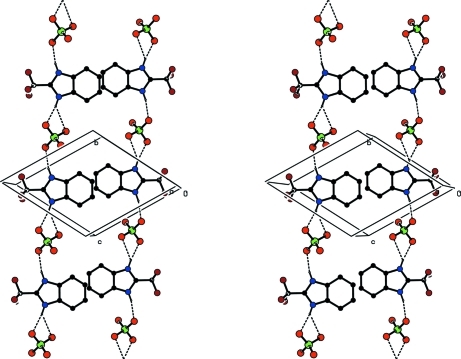
A stereoview of part of the crystal structure of compound showing the π–π linked cation-anion hydrogen-bonded ribbons. Hydrogen atoms not involved in the motifs are not included.

## supplementary crystallographic information

### Comment

Some interesting physical properties of simple molecular–ionic crystals containing organic cations and anions have been discussed by several authors (Yu *et al.*, 2004; Chen *et al.*, 2009; Ge *et al.*, 2007).

The asymmetric unit of the present compound , C_8_H_6_N_2_H_6_F_3_^+^ ClO_4_^-^ consists of one 2-trifluoromethyl-1*H*-benzimidazole cation and one perchlorate anion, Figure 1.

The atoms of the benzimidazole ring (including the H atoms) are nearly co-planar with a rms deviation of the fitted atoms of 0.0122Å. Atom C8 lies out of this plane. The perchlorate anion is a distorted tetrahedron, the average C—O bond distances range from 1.412 (3)Å to 1.439 (2)Å, the O—C—O angles range from 107.71 (17)° to 110.65 (15)°.

The 2-trifluoromethyl-1*H*-benzimidazole cations are linked to adjacent anions by intermolecular N-H···O hydrogen bonds to form chains, Table 1, Figure 2. The hydrogen bond involving N2-H2A is a three-centre contact to atoms O2 and O3 in which the H2A···O2, 2.05Å, is shorter than the H2A···O3, 2.59Å with the angles at H2A being 164° and 137° respectively. Centrosymmetrically related chains run through each unit cell and these are linked into ribbons by a π–π interaction between the phenyl rings, Cg1···Cg1(-x,1-y,1-z), 4.039 (2)Å, perpendicular distance between rings, 3.5948 (14)Å and slippage, 1.841Å. These ribbons run perpendicular to the *a-axis*.

.

### Experimental

0.144 g(1 mmol) of 2-trifluoromethyl-1*H*-benzimidazole was firstly dissolved in 30 ml methanol, to which 0.1 g (1 mmol) of perchloric acid was added to give a solution without any precipitate while stirring at the ambient temperature. Single crystals suitable for X-ray structure analysis were obtained by the slow evaporation of the above solution after 2 days in air.

The dielectric constant of the compound as a function of temperature indicates that the permittivity is basically temperature-independent (*ε* = C/(T–T_0_)), suggesting that this compound is not ferroelectric or there may be no distinct phase transition occurring within the measured temperature within the measured temperature (below the melting point).

### Refinement

The asymmetric unit was selected to form a hydrogen-bonded unit. H atoms attached to C atoms were placed in calculated positions with C—H = 0.93Å with *U*_iso_ = 1.2*U*eq and allowed to ride. The H atoms attached to the N atoms were located on a difference map. The N—H distances were restrained to 0.86Å and refined as riding atoms with *U*_iso_ = 1.2*U*eq in the final cycles of refinement. The 2 0 0 reflection was omitted from the refinement because of extinction.

### Crystal data

**Table:**

C_8_H_6_F_3_N_2_^+^·ClO_4_^−^	*V* = 554.0 (3) Å^3^
*M**_r_* = 286.60	*Z* = 2
Triclinic, *P*1	*F*(000) = 288
Hall symbol: -P 1	*D*_x_ = 1.718 Mg m^−^^3^
*a* = 7.6274 (15) Å	Mo *K*α radiation, λ = 0.71073 Å
*b* = 9.0614 (18) Å	θ = 3.4–26°
*c* = 9.3838 (19) Å	µ = 0.40 mm^−^^1^
α = 61.80 (3)°	*T* = 293 K
β = 81.98 (3)°	Block, colourless
γ = 75.85 (3)°	0.36 × 0.32 × 0.28 mm

### Data collection

**Table:**

Rigaku Mercury2 diffractometer	2535 independent reflections
Radiation source: fine-focus sealed tube	1980 reflections with *I* > 2σ(*I*)
graphite	*R*_int_ = 0.026
CCD_Profile_fitting scans	θ_max_ = 27.5°, θ_min_ = 3.4°
Absorption correction: multi-scan (*CrystalClear*; Rigaku, 2005)	*h* = −9→9
*T*_min_ = 0.963, *T*_max_ = 0.971	*k* = −11→11
5756 measured reflections	*l* = −12→12

### Refinement

**Table:**

Refinement on *F*^2^	Primary atom site location: structure-invariant direct methods
Least-squares matrix: full	Secondary atom site location: difference Fourier map
*R*[*F*^2^ > 2σ(*F*^2^)] = 0.051	Hydrogen site location: inferred from neighbouring sites
*wR*(*F*^2^) = 0.140	H-atom parameters constrained
*S* = 1.04	*w* = 1/[σ^2^(*F*_o_^2^) + (0.0636*P*)^2^ + 0.384*P*] where *P* = (*F*_o_^2^ + 2*F*_c_^2^)/3
2535 reflections	(Δ/σ)_max_ < 0.001
163 parameters	Δρ_max_ = 0.49 e Å^−^^3^
0 restraints	Δρ_min_ = −0.34 e Å^−^^3^

### Special details

**Table:**

Geometry. All esds (except the esd in the dihedral angle between two l.s. planes) are estimated using the full covariance matrix. The cell esds are taken into account individually in the estimation of esds in distances, angles and torsion angles; correlations between esds in cell parameters are only used when they are defined by crystal symmetry. An approximate (isotropic) treatment of cell esds is used for estimating esds involving l.s. planes.
Refinement. Refinement of *F*^2^ against ALL reflections. The weighted *R*-factor *wR* and goodness of fit *S* are based on *F*^2^, conventional *R*-factors *R* are based on *F*, with *F* set to zero for negative *F*^2^. The threshold expression of *F*^2^ > σ(*F*^2^) is used only for calculating *R*-factors(gt) *etc*. and is not relevant to the choice of reflections for refinement. *R*-factors based on *F*^2^ are statistically about twice as large as those based on *F*, and *R*- factors based on ALL data will be even larger.

### Fractional atomic coordinates and isotropic or equivalent isotropic displacement parameters (Å^2^)

**Table:**

	*x*	*y*	*z*	*U*_iso_*/*U*_eq_	
F1	0.3287 (4)	0.0295 (2)	0.1487 (2)	0.0793 (7)	
F2	0.4027 (3)	0.2471 (3)	−0.0564 (2)	0.0697 (6)	
F3	0.1269 (3)	0.2305 (3)	−0.0094 (3)	0.0791 (7)	
N1	0.2447 (3)	0.4517 (3)	0.1052 (3)	0.0430 (5)	
H1A	0.2375	0.5249	0.0045	0.052*	
N2	0.2695 (3)	0.2099 (3)	0.3226 (2)	0.0379 (5)	
H2A	0.2802	0.1020	0.3852	0.046*	
C1	0.2548 (3)	0.3343 (3)	0.3721 (3)	0.0374 (5)	
C2	0.2378 (4)	0.4892 (3)	0.2328 (3)	0.0395 (6)	
C3	0.2214 (4)	0.6432 (4)	0.2384 (4)	0.0534 (7)	
H3	0.2090	0.7476	0.1453	0.064*	
C4	0.2248 (5)	0.6313 (4)	0.3883 (4)	0.0603 (8)	
H4	0.2148	0.7308	0.3976	0.072*	
C5	0.2429 (5)	0.4748 (4)	0.5284 (4)	0.0593 (8)	
H5	0.2448	0.4737	0.6277	0.071*	
C6	0.2577 (4)	0.3237 (4)	0.5248 (3)	0.0510 (7)	
H6	0.2691	0.2199	0.6185	0.061*	
C7	0.2639 (3)	0.2848 (3)	0.1636 (3)	0.0376 (5)	
C8	0.2812 (4)	0.1957 (4)	0.0614 (3)	0.0483 (7)	
Cl1	0.21788 (9)	0.83165 (8)	−0.28977 (7)	0.0426 (2)	
O1	0.3244 (3)	0.8661 (3)	−0.4364 (2)	0.0571 (6)	
O2	0.1589 (4)	0.6730 (3)	−0.2310 (3)	0.0692 (7)	
O3	0.3197 (4)	0.8220 (5)	−0.1699 (3)	0.0965 (10)	
O4	0.0612 (4)	0.9641 (3)	−0.3218 (3)	0.0806 (8)	

### Atomic displacement parameters (Å^2^)

**Table:**

	*U*^11^	*U*^22^	*U*^33^	*U*^12^	*U*^13^	*U*^23^
F1	0.131 (2)	0.0469 (11)	0.0617 (12)	−0.0124 (11)	−0.0017 (12)	−0.0287 (9)
F2	0.0740 (13)	0.0940 (15)	0.0507 (10)	−0.0281 (11)	0.0205 (9)	−0.0414 (10)
F3	0.0649 (12)	0.1233 (19)	0.0777 (14)	−0.0174 (12)	−0.0104 (10)	−0.0676 (14)
N1	0.0551 (13)	0.0378 (11)	0.0265 (10)	−0.0127 (10)	0.0002 (9)	−0.0058 (9)
N2	0.0466 (12)	0.0319 (10)	0.0273 (10)	−0.0068 (9)	−0.0029 (9)	−0.0071 (8)
C1	0.0396 (13)	0.0364 (13)	0.0318 (12)	−0.0081 (10)	−0.0015 (10)	−0.0118 (10)
C2	0.0442 (14)	0.0383 (13)	0.0316 (12)	−0.0115 (11)	−0.0005 (10)	−0.0112 (10)
C3	0.068 (2)	0.0385 (14)	0.0502 (17)	−0.0166 (14)	−0.0008 (14)	−0.0149 (13)
C4	0.076 (2)	0.0521 (18)	0.065 (2)	−0.0212 (16)	0.0012 (17)	−0.0337 (16)
C5	0.076 (2)	0.068 (2)	0.0453 (16)	−0.0156 (17)	−0.0024 (15)	−0.0339 (16)
C6	0.0646 (18)	0.0515 (16)	0.0323 (13)	−0.0109 (14)	−0.0049 (12)	−0.0149 (12)
C7	0.0389 (13)	0.0394 (13)	0.0291 (12)	−0.0098 (10)	0.0010 (10)	−0.0111 (10)
C8	0.0560 (17)	0.0536 (16)	0.0374 (14)	−0.0143 (13)	0.0025 (12)	−0.0219 (13)
Cl1	0.0500 (4)	0.0392 (3)	0.0302 (3)	−0.0106 (3)	−0.0028 (2)	−0.0079 (2)
O1	0.0636 (13)	0.0512 (12)	0.0351 (10)	−0.0059 (10)	0.0047 (9)	−0.0067 (9)
O2	0.0965 (18)	0.0503 (13)	0.0569 (14)	−0.0310 (12)	0.0130 (13)	−0.0178 (11)
O3	0.091 (2)	0.158 (3)	0.0524 (14)	−0.057 (2)	−0.0063 (14)	−0.0410 (17)
O4	0.0728 (16)	0.0586 (15)	0.0820 (18)	0.0036 (12)	0.0105 (14)	−0.0207 (13)

### Geometric parameters (Å, °)

**Table:**

F1—C8	1.312 (4)	C3—C4	1.363 (4)
F2—C8	1.317 (3)	C3—H3	0.9300
F3—C8	1.328 (4)	C4—C5	1.397 (5)
N1—C7	1.318 (3)	C4—H4	0.9300
N1—C2	1.382 (3)	C5—C6	1.362 (4)
N1—H1A	0.86	C5—H5	0.9300
N2—C7	1.318 (3)	C6—H6	0.9300
N2—C1	1.385 (3)	C7—C8	1.495 (4)
N2—H2A	0.86	Cl1—O3	1.412 (3)
C1—C2	1.385 (3)	Cl1—O4	1.420 (3)
C1—C6	1.393 (4)	Cl1—O1	1.434 (2)
C2—C3	1.395 (4)	Cl1—O2	1.439 (2)
			
C7—N1—C2	108.6 (2)	C4—C5—H5	118.8
C7—N1—H1A	125.7	C5—C6—C1	115.8 (3)
C2—N1—H1A	125.8	C5—C6—H6	122.1
C7—N2—C1	108.5 (2)	C1—C6—H6	122.1
C7—N2—H2A	125.8	N2—C7—N1	110.3 (2)
C1—N2—H2A	125.7	N2—C7—C8	125.6 (2)
N2—C1—C2	106.3 (2)	N1—C7—C8	124.1 (2)
N2—C1—C6	131.7 (2)	F1—C8—F2	108.7 (3)
C2—C1—C6	121.9 (3)	F1—C8—F3	108.6 (3)
N1—C2—C1	106.3 (2)	F2—C8—F3	106.2 (2)
N1—C2—C3	132.0 (2)	F1—C8—C7	110.7 (2)
C1—C2—C3	121.6 (3)	F2—C8—C7	111.0 (2)
C4—C3—C2	116.0 (3)	F3—C8—C7	111.4 (2)
C4—C3—H3	122.0	O3—Cl1—O4	110.0 (2)
C2—C3—H3	122.0	O3—Cl1—O1	110.25 (16)
C3—C4—C5	122.2 (3)	O4—Cl1—O1	109.06 (15)
C3—C4—H4	118.9	O3—Cl1—O2	109.08 (18)
C5—C4—H4	118.9	O4—Cl1—O2	107.71 (17)
C6—C5—C4	122.4 (3)	O1—Cl1—O2	110.65 (15)
C6—C5—H5	118.8		
			
C7—N2—C1—C2	0.7 (3)	N2—C1—C6—C5	178.7 (3)
C7—N2—C1—C6	−178.2 (3)	C2—C1—C6—C5	0.0 (4)
C7—N1—C2—C1	0.2 (3)	C1—N2—C7—N1	−0.6 (3)
C7—N1—C2—C3	179.0 (3)	C1—N2—C7—C8	178.0 (2)
N2—C1—C2—N1	−0.5 (3)	C2—N1—C7—N2	0.2 (3)
C6—C1—C2—N1	178.5 (3)	C2—N1—C7—C8	−178.4 (2)
N2—C1—C2—C3	−179.4 (2)	N2—C7—C8—F1	−9.0 (4)
C6—C1—C2—C3	−0.5 (4)	N1—C7—C8—F1	169.4 (3)
N1—C2—C3—C4	−178.0 (3)	N2—C7—C8—F2	−129.8 (3)
C1—C2—C3—C4	0.6 (4)	N1—C7—C8—F2	48.6 (4)
C2—C3—C4—C5	−0.2 (5)	N2—C7—C8—F3	112.1 (3)
C3—C4—C5—C6	−0.2 (6)	N1—C7—C8—F3	−69.5 (3)
C4—C5—C6—C1	0.3 (5)		

### Hydrogen-bond geometry (Å, °)

**Table:**

*D*—H···*A*	*D*—H	H···*A*	*D*···*A*	*D*—H···*A*
N1—H1A···O2	0.86	2.05	2.891 (3)	164
N1—H1A···O3	0.86	2.59	3.254 (4)	135
N2—H2A···O1^i^	0.86	1.98	2.822 (3)	167

Symmetry codes: (i) *x*, *y*−1, *z*+1.
